# Association between skin sympathetic nerve activity and electrocardiogram alterations after subarachnoid hemorrhage

**DOI:** 10.14814/phy2.70202

**Published:** 2025-01-14

**Authors:** Yoichiro Nakagawa, Takashi Kusayama, Sho Tamai, Yuta Nagamori, Kazutaka Takeuchi, Shuhei Iwaisako, Taishi Tsutsui, Tomoya Kamide, Kouichi Misaki, Soichiro Usui, Kenji Sakata, Mitsutoshi Nakada, Masayuki Takamura

**Affiliations:** ^1^ Department of Cardiovascular Medicine Kanazawa University Graduate School of Medical Sciences Kanazawa Ishikawa Japan; ^2^ Department of Emergency and Disaster Medicine Kanazawa University Graduate School of Medical Sciences Kanazawa Ishikawa Japan; ^3^ Department of Neurosurgery Kanazawa University Graduate School of Medical Sciences Kanazawa Ishikawa Japan; ^4^ Department of Health Sciences Kanazawa University Graduate School of Medical Sciences Kanazawa Ishikawa Japan

**Keywords:** brain–heart interaction, electrocardiogram, repolarization abnormality, skin sympathetic nerve activity, subarachnoid hemorrhage

## Abstract

While autonomic dysregulation and repolarization abnormalities are observed in subarachnoid hemorrhage (SAH), their relationship remains unclear. We aimed to measure skin sympathetic nerve activity (SKNA), a novel method to estimate stellate ganglion nerve activity, and investigate its association with electrocardiogram (ECG) alterations after SAH. We recorded a total of 179 SKNA data from SAH patients at three distinct phases and compared them with 20 data from controls. Compared with control data, in the acute phase data (days 1–3 of SAH), T peak‐to‐end (Tp‐e) interval was significantly prolonged (81 [interquartile range {IQR}: 71–93] vs. 58 [IQR: 54–64] ms, *p* < 0.001), non‐burst amplitude of SKNA was significantly increased (2.4 [IQR: 1.3–4.1] vs. 0.7 [IQR: 0.5–1.7] μV, *p* < 0.001), and the ratio of low frequency to high frequency (HF) was significantly decreased (0.8 [IQR: 0.5–1.1] vs. 1.1 [IQR: 0.7–1.3], *p* = 0.028). Linear mixed model revealed a relationship between Tp‐e interval and SKNA. Although these abnormalities gradually normalized, delayed cerebral ischemia episodes were associated with increased HF oscillation. Transient sympathetic dysregulation contributes to repolarization impairment after SAH. SKNA may have the potential to monitor adverse outcomes.

## 1 INTRODUCTION

Subarachnoid hemorrhage (SAH) caused by aneurysm rupture has the poorest outcome among strokes (Roos et al., 2000). Electrocardiogram (ECG) abnormalities after SAH are known (Kawasaki et al., 2002), with QT interval prolongation and ST changes linked to a worse prognosis (Elsharkawy et al., 2016; Takenaka et al., 2006). Similarly, T peak‐to‐end (Tp‐e) interval prolongation, indicating arrhythmia risk, correlates with neurological severity (Komatsuzaki et al., 2021). Studies show arrhythmias develop in up to 4.3% of SAH patients within 3 days after onset, likely due to increased sympathetic nerve activity (SNA) (Frontera et al., 2008; Marion et al., 1986). However, the mechanisms and course of these ECG alterations remain unclear. Furthermore, while the onset of delayed cerebral ischemia (DCI) after SAH significantly worsens the neurological prognosis, its pathology is complex, and early detection and establishment of treatment methods are challenges (Vergouwen et al., 2010).

The stellate ganglion (SG) is an important node of sympathetic nerves that regulate various cardiovascular functions, and the SNA originated from the SG is defined as SG nerve activity (SGNA). It is known to be excessively enhanced SNA during the acute phase of SAH (Demura et al., 2023), and blockade of SG may prevent DCI (Jain et al., 2011). Recognizing the close relationship between the brain and heart (i.e., brain–heart interaction) (Battaglini et al., 2020), Chen et al. (2023) demonstrated in a rat model that the QT interval prolongation and SGNA peak on the third day after SAH, then gradually normalize in the later phases (Chen et al., 2023). However, the mechanism that causes ECG alterations after SAH is not fully understood, and no studies have directly investigated the relationship between ECG alterations and SNA after SAH in humans. neuECG is a novel noninvasive method that was developed to allow simultaneous recording of ECG and skin SNA (SKNA) in humans (Doytchinova et al., 2017; Kusayama et al., 2020). As described in previous studies, SGNA can be estimated by measuring SKNA of the chest and upper extremities (Doytchinova et al., 2017; Jiang et al., 2015; Taniguchi et al., 1994). Heart rate variability (HRV) is another noninvasive method for estimating cardiac SNA. However, HRV lacks the temporal resolution to monitor SNA and is challenging to measure during sustained arrhythmias such as atrial fibrillation. Since neuECG allows simultaneous monitoring of ECG and SKNA, we thought it could contribute to the early detection of neurological complications and arrhythmia events that determine the prognosis of SAH. The present study aims to explore the possibility of clinical application as a pilot study by testing the hypothesis that SKNA is associated with ECG alterations after SAH and to clarify the time course of these parameters in humans.

## 2 METHODS

### 2.1 Ethics statement

All procedures in the present study were performed according to ethical standards. The present study was approved by the local bioethical committee of Kanazawa University (113858) and was performed in accordance with the Declaration of Helsinki of the World Medical Association.

### 2.2 Participants

**FIGURE 1:**
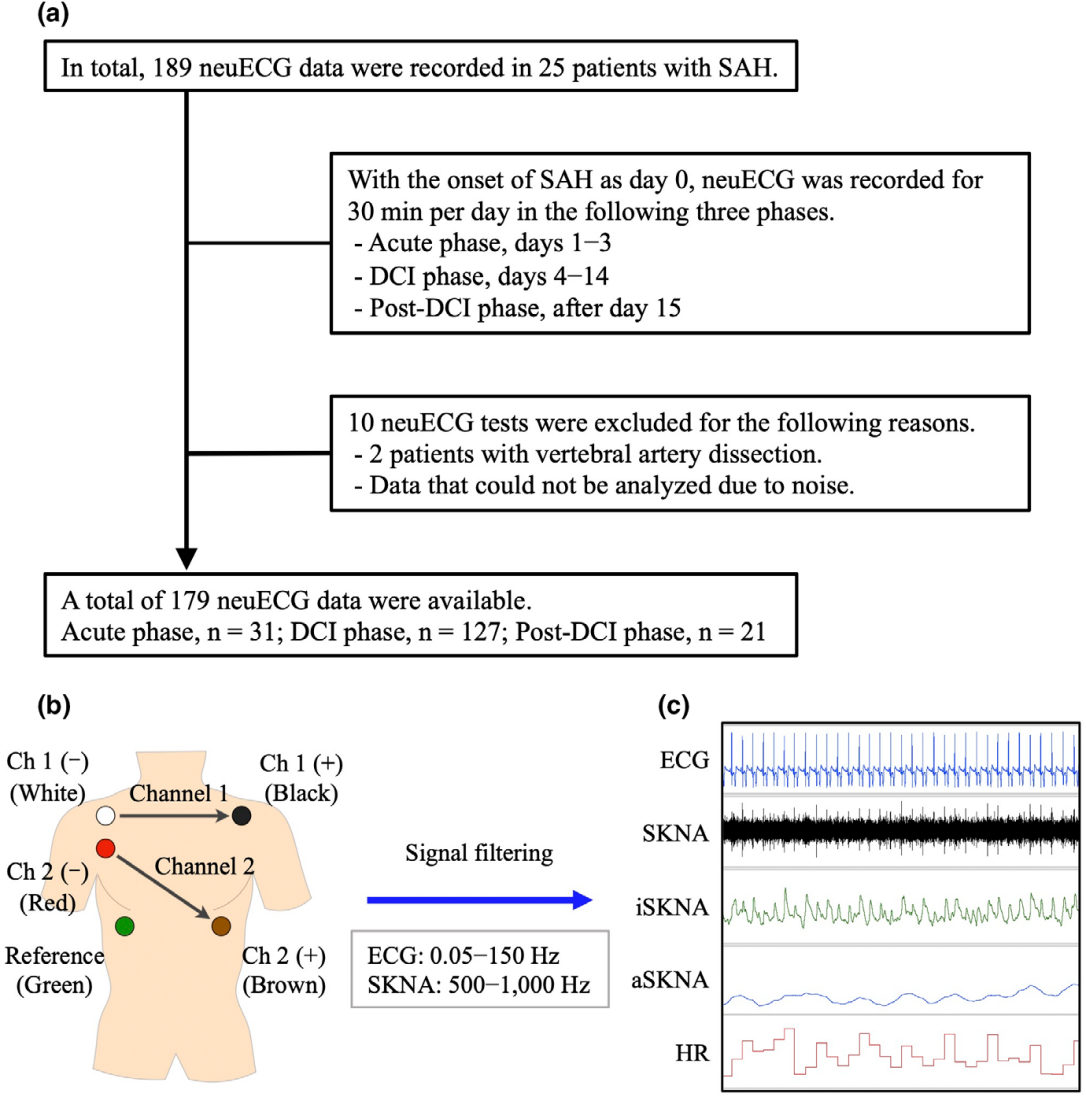
Study protocol. (a) We obtained a total of 189 neuECG tests from 25 patients diagnosed with subarachnoid hemorrhage (SAH). The day of onset was designated as day 0, and neuECG was recorded in three distinct phases, as follows: Acute phase, days 1–3 (*n* = 31); delayed cerebral ischemia (DCI) phase, days 4–14 (*n* = 127); and post‐DCI phase, after day 15 (*n* = 21). (b) Schematic illustration of electrode patch locations. Channel (Ch) 1 was recorded from the negative electrode (white) placed in the right subclavian area to the positive electrode (black) in the left side. Ch 2 was recorded from the negative electrode (red) placed in the right subclavian area to the positive electrode (brown) in the left abdomen. The green electrode served as a reference. neuECG recordings were band‐pass filtered and displayed at 0.05–150 Hz for electrocardiogram (ECG) and 500–1000 Hz for skin sympathetic nerve activity (SKNA). (c) Each SKNA sample was integrated (iSKNA) or averaged (aSKNA) into analyzable data and displayed.

We analyzed 25 patients diagnosed with spontaneous SAH who were admitted to Kanazawa University Hospital between March 2022 and June 2023 (Figure 1a). Two patients with non‐aneurysmal SAH (vertebral artery dissection) were excluded. The diagnosis of SAH was confirmed via computed tomography (CT) scan. To evaluate cerebral vasospasm and diagnose cerebral infarction during DCI, magnetic resonance imaging (MRI) was performed. For comparison, 20 subjects who were scheduled for catheter ablation for paroxysmal supraventricular tachycardia without other problems were also included as controls. Before enrollment, informed consent was obtained from all participants or their representatives using a written document approved by our institutional ethics committee. All participants were aged ≥18 years and had no history of neurological diseases.

### 2.3 Treatment and management of SAH

All patients received guideline‐directed treatment. In each case, immediate coil embolization or clipping surgery under general anesthesia was performed to prevent rebleeding. During the acute stage, each patient was discontinued sedatives and ventilator‐based respiratory management as early as possible, depending on the patient's general condition. Adequate blood pressure and intravascular volume maintenance and standard drug administration were performed to prevent the development of DCI and cerebral vasospasm. Continuous ECG monitoring was performed to detect arrhythmia events.

### 2.4 Study protocol

The day of onset of SAH was designated as day 0, and neuECG was recorded in three distinct phases, as follows: acute phase, days 1–3; DCI phase, days 4–14; and post‐DCI phase, after day 15. Each recording was conducted for 30 min per day when the patient was able to maintain bed rest. Using neuECG data obtained repeatedly in each phase, changes in ECG, HRV, and SKNA parameters were compared with the control data. Furthermore, to detect abnormalities when a DCI episode occurred, during the DCI phase, DCI recordings were defined as neuECG recordings conducted on the same day as the DCI episodes, and non‐DCI recordings were defined as those not associated with DCI episodes. DCI was defined as the new onset of focal neurological impairment or overall neurological deterioration (a decrease in the Glasgow Coma Scale [GCS] score to >2) that lasted for 1 h after the exclusion of intracranial hemorrhage, hydrocephalus, seizures, metabolic derangements, and infection, with or without the presence of cerebral vasospasm on radiological imaging (Vergouwen et al., 2010).

### 2.5 neuECG recording and SKNA analysis

In the present study, neuECG recordings were performed according to a previously reported experimental protocol in humans, and SGNA was estimated by measuring SKNA in the chest and upper extremities (Kusayama et al., 2020) (Figure 1b). Sympathetic postganglionic fibers from the canine SG have been shown to be distributed in the chest and upper extremities, a skin area between the level of the ipsilateral third cervical vertebra and the thirteenth rib (Taniguchi et al., 1994). SKNA is clearly distinct from skin sympathetic nerve activity (SSNA) using microneurography. SSNA directly observes the activity of skin nerve fascicles, and while it has been shown to have a deep relationship with blood flow and temperature regulation, it is invasive and has minimal involvement with blood pressure or heart beats (Hart et al., 2017). On the contrary, SKNA has been confirmed to have a close correlation with SGNA (Jiang et al., 2015), and may more sensitively reflect the relationship between neural activity in the heart and brain. SKNA signals were managed by the LabChart software (ADInstruments Ltd., Bella Vista, Australia), with a sampling rate of 10 k/s (Liu et al., 2021). Channel 2 was used, and the extracted data were manually inspected for 30 min to exclude periods of motion artifacts. SKNA signals have a frequency range from 0 to >2000 Hz and amplitudes ranging from 0.5 to 100 μV in most cases. To distinguish SKNA signals from other electrical sources, such as ECG and myopotential signals, filtering is employed. ECG recordings have amplitudes measured in millivolts with frequency content ranging from 0.05 to 150 Hz in adults (Kligfield & Okin, 2007). Myopotential signals, or the electrical activations from the skeletal muscle, can also reach millivolts and have a frequency content as high as 400 Hz, although most are <100 Hz (Komi & Tesch, 1979). By applying a 500‐Hz high‐pass filter, ECG and myopotential signals can be eliminated, preserving the nerve activity. Additionally, a 1000‐Hz low‐pass filter minimizes contamination by high‐frequency electrical noise commonly present in patient's care areas (Figure 1b). The average SKNA (aSKNA) was defined as the mean voltage of each signal (Figure 1c). Fast Fourier transform (FFT) analyses of the integrated SKNA (iSKNA) were performed to collect frequency components (very low frequency [VLF]: 0.01–0.04 Hz, low frequency [LF]: 0.04–0.15 Hz, and high frequency [HF]: 0.15–0.40 Hz) (Kusayama et al., 2020; Meng et al., 2022). The signals were downsampled from 10 k/s to 1 k/s before using FFT analyses with an FFT size of 128 k and an overlap of 93.75% to improve the frequency resolution. The normalization of the power of each frequency band was divided by the total power of the signals to obtain different frequency components (VLFnu, LFnu, and HFnu [normalized unit {nu}]). The dominant frequency was the frequency with the highest power in 30 min.

### 2.6 ECG and HRV analysis

ECG and HRV analysis were also performed using the LabChart software. Averaged ECG data from neuECG were employed, with 1 min of data used for ECG analysis and 30 min for HRV analysis. To ensure the accuracy of the analysis, a total of three recordings were excluded, including those affected by motion artifacts and those that posed challenges to the ECG analysis due to atrial fibrillation or frequent ectopic beats. The ECG alterations involved PR intervals, corrected QT (QTc) intervals, and Tp‐e intervals. The QT intervals were calculated using the Bazett method. In HRV analysis, each calculated frequency component (VLF: 0.01–0.04 Hz, LF: 0.04–0.15 Hz, and HF: 0.15–0.40 Hz) was divided by the total power frequency to divide it into the normalized components (VLF_nu‐HRV_, LF_nu‐HRV_, and HF_nu‐HRV_). The HF component reflects vagal modulation, while the LF component represents a combination of sympathetic and parasympathetic influences (Tiwari et al., 2021). Understanding the relative contributions of HF and LF allows for a more nuanced interpretation of autonomic nervous system modulation.

### 2.7 Statistical analysis

Data with non‐normal distribution were presented as median (interquartile range [IQR]). Continuous variables between unpaired data were compared using the Wilcoxon rank‐sum test. Pearson's chi‐square test was conducted to compare categorical variables. Steel's multiple comparison test was used when comparing variables between the control group and multiple study groups. Linear mixed models were used to assess the relationship between ECG alterations and SKNA parameters while accounting for the repeated measurements per patient. All statistical analyses were conducted using JMP Pro version 14 (SAS Institute, Cary, NC, the USA), and all figures were created using GraphPad Prism 9 (GraphPad Software, Inc., La Jolla, the USA). Two‐sided *p* values of <0.05 were considered statistically significant. Post hoc power of the study was estimated using the G*Power (version 3.1.9.7).

## 3 RESULTS

### 3.1 Characteristics of the participants

Table 1 shows the characteristics of the participants. Although there were no differences in age or sex between the patients with SAH (*n* = 23) and the control subjects (*n* = 20), the proportion of smokers was significantly higher in the former. At the onset of SAH, GCS scores were 3–6; 6 (26.1%), 7–10; 5 (21.7%), 11–15; 12 (52.2%), and Hunt and Kosnik grades were 1–2; 4 (17.4%), 3–4; 15 (65.2%), and 5; 4 (17.4%). Compared with the control subjects, the patients with SAH had significantly lower serum levels of K (4.1 [IQR: 3.9–4.4] vs. 3.7 [IQR: 3.4–3.9] mEq/L, *p* = 0.001), Ca (9.1 [IQR: 8.8–9.5] vs. 8.3 [IQR: 8.0–9.0] mg/dL, *p* = 0.004), and Mg (2.1 [IQR: 1.9–2.1] vs. 1.9 [IQR: 1.8–2.0] mg/dL, *p* = 0.007) at hospitalization. Regarding the ECG abnormalities, the frequency of negative T waves was significantly higher in the patients with SAH than in the control subjects (9 [42.9%] vs. 2 [10.0%], *p* = 0.017). In patients with SAH, no ventricular arrhythmia events were observed, and 2 (8.7%) had atrial fibrillation. On the contrary, the two groups had no differences in the frequencies of atrioventricular block or bundle branch block and in the use of drugs that can affect the QT interval, such as antidepressants, antiarrhythmic, and macrolide and quinolone antibiotics. The majority of ruptured aneurysms were located in the middle cerebral artery (8 patients, 34.8%) and the anterior communicating artery (8 patients, 34.8%) (Table 2). There was no significant laterality in the affected hemisphere, and the majority of patients presented with bilateral hematoma. A total of six patients (26.1%) developed DCI during the clinical course.

**TABLE 1:** Characteristics of the participants.

Variables	Control subjects (*n* = 20)	Patients with SAH (*n* = 23)	*p* Value
Age (years)	64 (39–69)	60 (55–76)	0.324
Male sex, *n* (%)	11 (55.0)	7 (30.4)	0.103
HT, *n* (%)	7 (35.0)	6 (26.1)	0.526
DL, *n* (%)	8 (40.0)	7 (30.4)	0.512
Smoking, *n* (%)	0 (0.0)	7 (30.4)	0.007
Blood tests			
Ht (%)	41 (38–45)	41 (37–45)	0.679
eGFR (mL/min/1.73m^2^)	82 (70–90)	79 (64–96)	0.952
Na (mEq/L)	142 (139–144)	141 (140–143)	0.523
K (mEq/L)	4.1 (3.9–4.4)	3.7 (3.4–3.9)	0.001
Ca (mg/dL)	9.1 (8.8–9.5)	8.3 (8.0–9.0)	0.004
Mg (mg/dL)	2.1 (1.9–2.1)	1.9 (1.8–2.0)	0.007
CK (IU/L)	100 (65–160)	112 (73–182)	0.742
ECG abnormalities			
Atrioventricular block, *n* (%)	2 (10.0)	0 (0.0)	0.120
Complete right bundle block, *n* (%)	3 (15.0)	1 (4.4)	0.230
Negative T wave, *n* (%)	2 (10.0)	9 (42.9)	0.017
AF, *n* (%)	0 (0.0)	2 (8.7)	0.177
VT/VF, *n* (%)	0 (0.0)	0 (0.0)	NA
Medications			
β blockers, *n* (%)	0 (0.0)	2 (8.7)	0.177
CCBs, *n* (%)	3 (15.0)	4 (17.4)	0.832
RASIs, *n* (%)	3 (15.0)	4 (17.4)	0.832
Statins, *n* (%)	3 (15.0)	4 (17.4)	0.832
Antidepressants, *n* (%)	0 (0.0)	2 (8.7)	0.177
Antiarrhythmic drugs, *n* (%)	1 (5.0)	1 (4.4)	0.919
Antibiotics, *n* (%)	0 (0.0)	0 (0.0)	NA

*Note*: Values in the table indicate the median (interquartile range) or total number (%).

Abbreviations: AF, atrial fibrillation; CCB, calcium channel blocker; CK, creatine kinase; DL, dyslipidemia; ECG, electrocardiogram; eGFR, estimated glomerular filtration rate; Ht, hematocrit; HT, hypertension; NA, not available; RASI, renin‐angiotensin system inhibitor; SAH, subarachnoid hemorrhage; VF, ventricular fibrillation; VT, ventricular tachycardia.

**TABLE 2:** Details in patients with SAH.

Variables	Patients with SAH (*n* = 23)
Localization of the ruptured aneurysm	
Middle cerebral artery, *n* (%)	8 (34.8)
Anterior communicating artery, *n* (%)	8 (34.8)
Internal carotid artery, *n* (%)	2 (8.7)
Posterior cerebral artery, *n* (%)	3 (13.0)
Basilar artery, *n* (%)	2 (8.7)
Localization of mainly affected hemisphere	
Right hemisphere, *n* (%)	5 (21.7)
Left hemisphere, *n* (%)	4 (17.4)
No difference, *n* (%)	14 (60.9)
Events after SAH	
DCI, *n* (%)	6 (26.1)
CVS, *n* (%)	9 (42.9)
Hydrocephalus, *n* (%)	11 (47.8)

*Note*: Values in the table indicate total number (%).

Abbreviations: CVS, cerebral vasospasm; DCI, delayed cerebral ischemia; SAH, subarachnoid hemorrhage.

### 3.2 Effects of SAH on ECG and its time course

**FIGURE 2:**
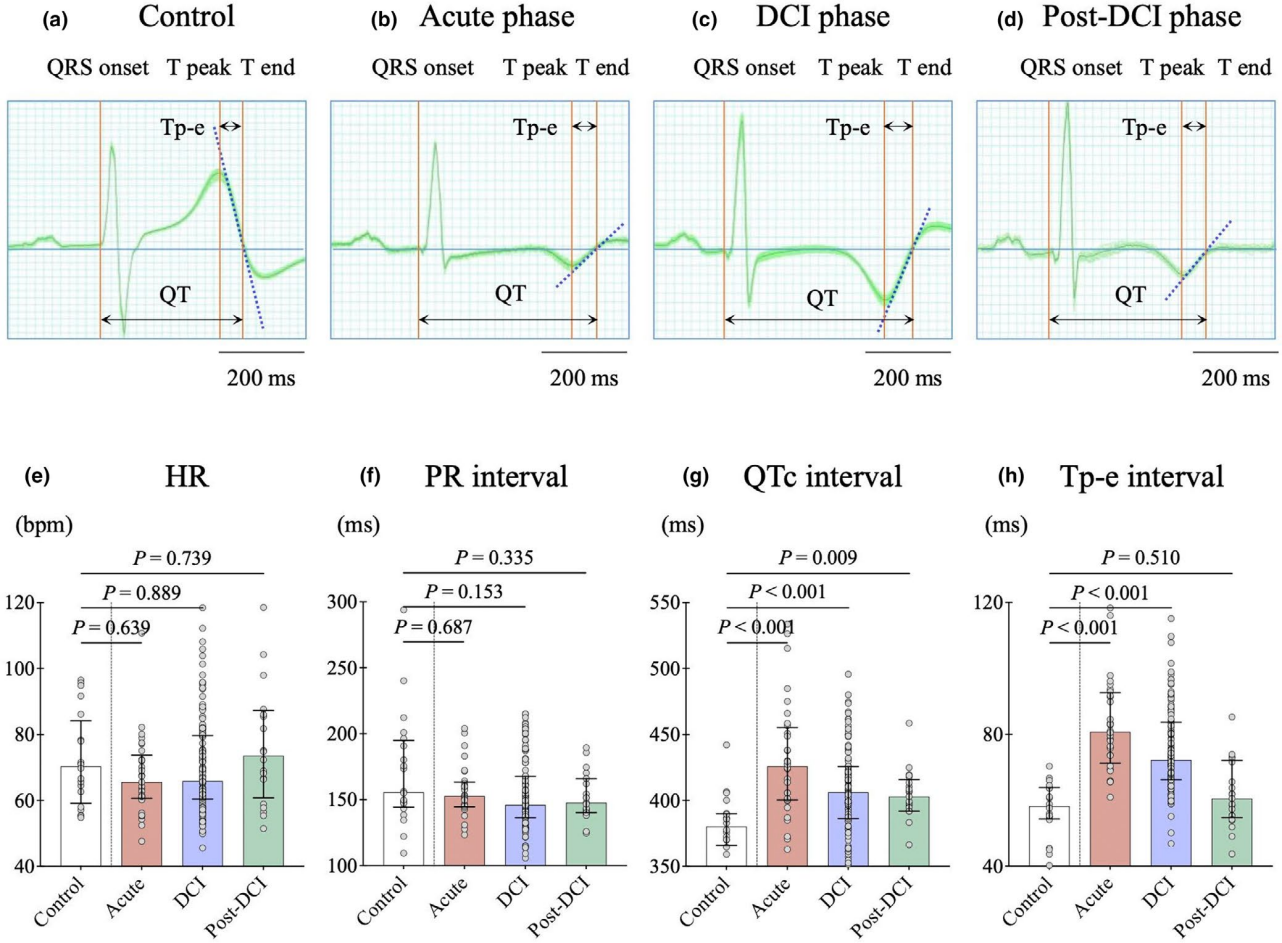
Effects of subarachnoid hemorrhage (SAH) on electrocardiogram (ECG) and its time course. (a–d) Representative average ECG waveforms of the control (*n* = 20) and each SAH phase. The SAH data were characterized by prominent negative T waves and prolonged corrected QT (QTc) and T peak‐to‐end (Tp‐e) intervals. These changes showed gradual improvement toward the post‐delayed cerebral ischemia (DCI) phase. (e, f) The dots in the figure represent each SKNA data, and the solid lines represent the median and quartiles. The heart rate (HR) and the PR interval were not significantly different between the SAH phases and the control data (*n* = 20), and no significant fluctuations were observed during the clinical course. (g, h) The QTc and Tp‐e intervals were significantly longer in the acute phase (*n* = 31). In the post‐DCI phase (*n* = 21), the improvement of the QTc interval was delayed compared to the Tp‐e interval.

A total of 189 neuECG data were recorded from patients with SAH, and a total of 10 data were excluded as excessive noise or frequent arrhythmia (3 data) and non‐aneurysmal SAH (7 data) (Figure 1a). The 179 available data included acute phase (*n* = 31), DCI phase (*n* = 127), and post‐DCI phase (*n* = 21) and were compared with control data (*n* = 20). Figure 2 shows the effects of SAH on ECG and its time course. Figure 2a–d show representative data. Compared with the control subjects, patients with SAH had prominent negative T waves and prolonged QTc and Tp‐e intervals in the acute phase; these changes gradually improved in the post‐DCI phase. The heart rate (HR) and the PR interval during the three phases were not significantly different, compared with the control data, and did not significantly fluctuate during the clinical course (Figure 2e,f). Compared with the control data, the acute phase was characterized by significantly longer QTc (426 [IQR: 400–455] vs. 380 [IQR: 366–390] ms, *p* < 0.001) and Tp‐e (81 [IQR: 71–93] vs. 58 [IQR: 54–64] ms, *p* < 0.001) intervals (Figure 2g,h). The QTc interval remained prolonged with delayed improvement during the entire course of SAH, whereas the Tp‐e interval gradually shortened toward the post‐DCI phase.

### 3.3 Effects of SAH on SKNA and HRV and its time course

**FIGURE 3:**
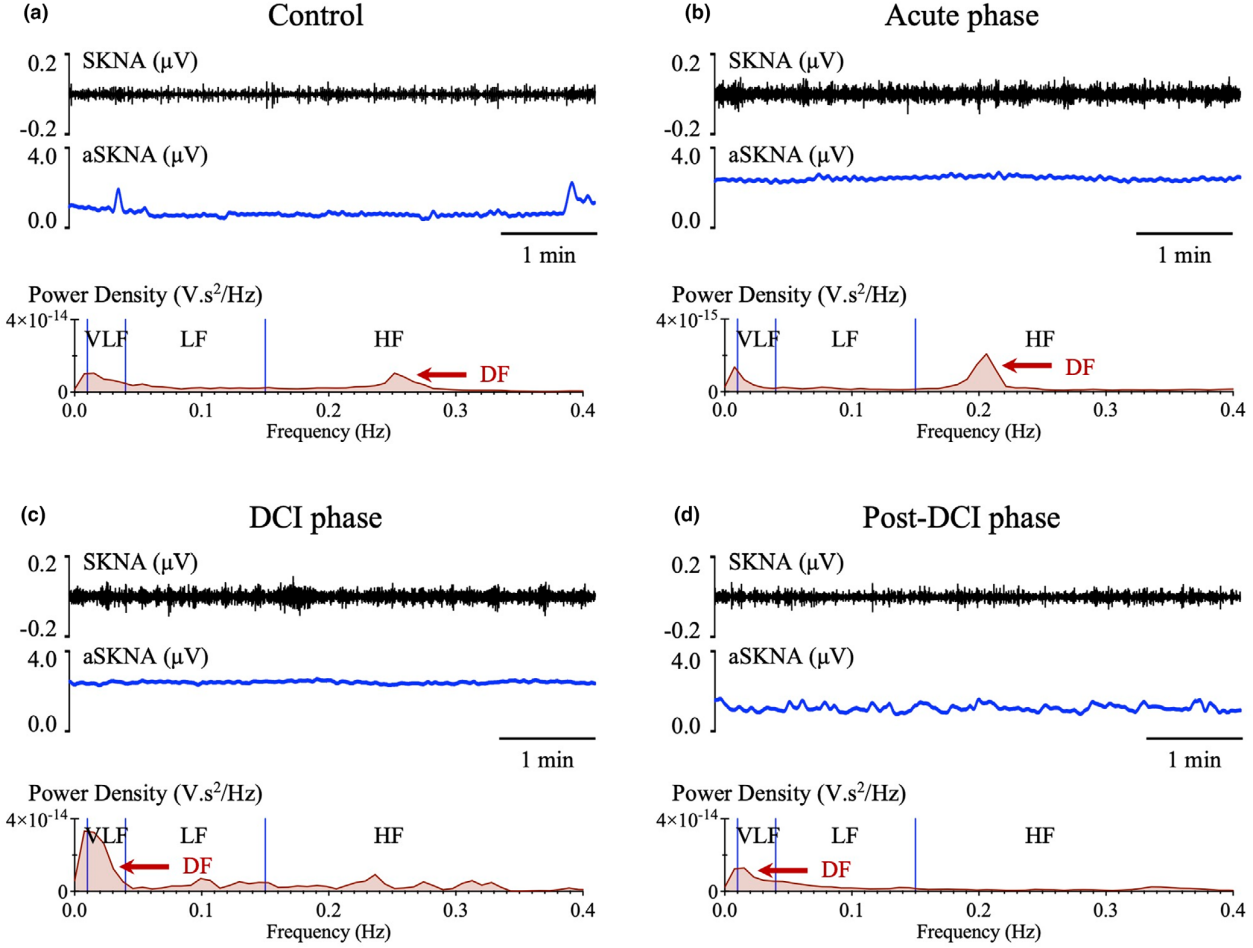
Representative skin sympathetic nerve activity (SKNA) and average SKNA (aSKNA) waveforms and frequency distributions in the three subarachnoid hemorrhage (SAH) phases. Compared to the (a) control data, the SKNA amplitude is enhanced during the (b) acute phase, with the high frequency (HF) band components becoming prominent. (c) During the delayed cerebral ischemia (DCI) phase, the SKNA amplitude remains enhanced, but the dominant frequency (DF) is shifted to the very low frequency (VLF) band. (d) During the post‐DCI phase, the SKNA amplitude is attenuated, and the frequency distribution is more homogeneous.

The representative data are presented as Figure 3. Compared to the control data, the SKNA amplitude is enhanced during the acute phase, with HF band components becoming prominent (Figure 3b). During the DCI phase, the SKNA amplitude remains enhanced, but the DF is shifted to the VLF band (Figure 3c). During the post‐DCI phase, the SKNA amplitude is attenuated, and the frequency distribution is more homogeneous (Figure 3d).

**FIGURE 4:**
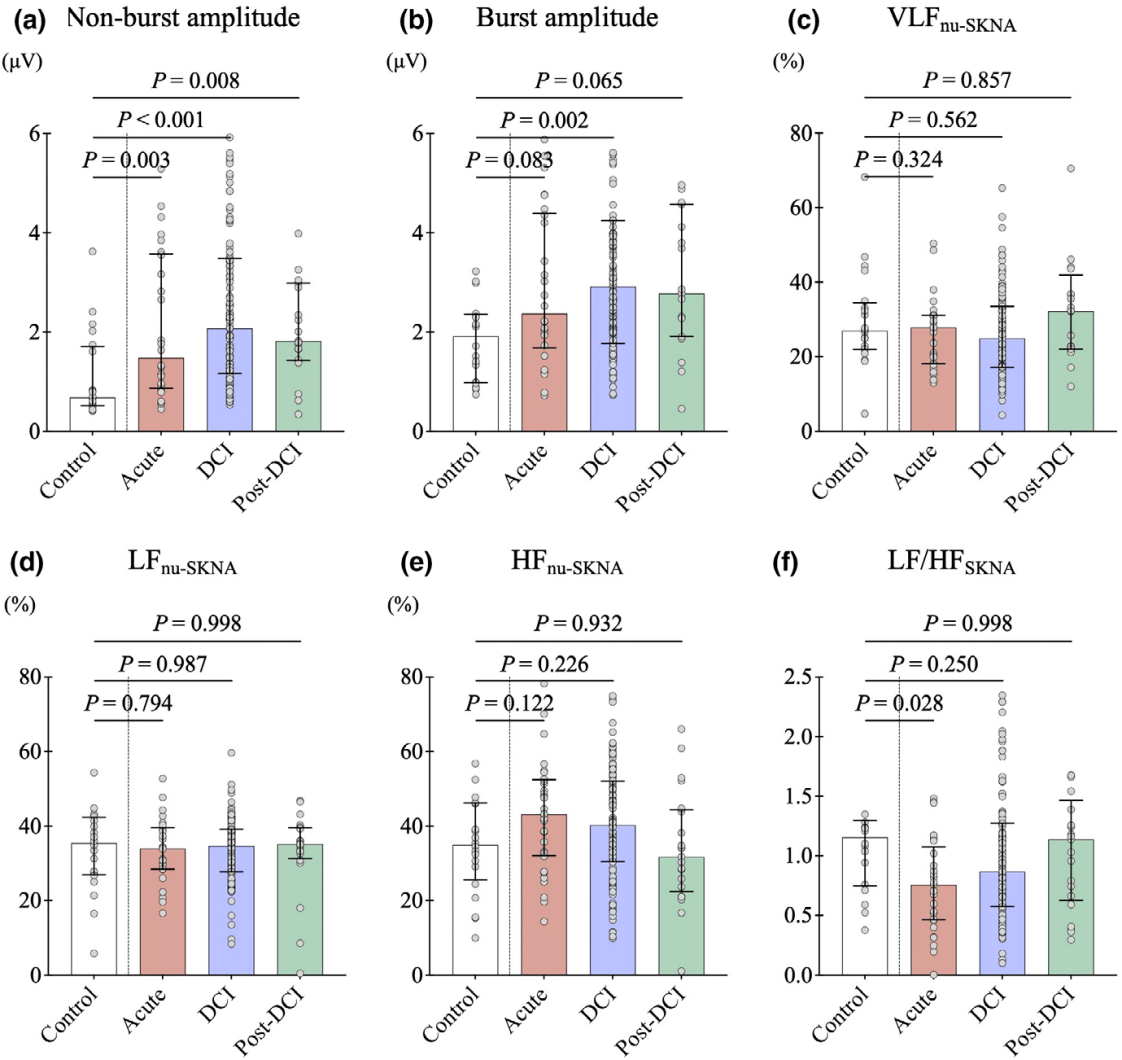
Effects of subarachnoid hemorrhage (SAH) on skin sympathetic nerve activity (SKNA) and its time course. The dots in the figure represent each SKNA data, and the solid lines represent the median and quartiles. In the SKNA analysis, there were significant enhancements of the average SKNA (aSKNA) (a) non‐burst amplitude and (b) burst amplitude in the acute phase (*n* = 31), reached a peak during the DCI phase (*n* = 127), and then gradually attenuated toward the post‐DCI phase (*n* = 21). (c–f) Compared with the control data (*n* = 20), the acute phase showed a significantly decreased ratio of low frequency to high frequency (LF/HF_SKNA_). The other three phases had no significant differences in any of the frequency components (i.e., very low frequency oscillation [VLF], low frequency oscillation [LF], and high frequency oscillation [HF]). However, in the acute phase, the proportion of HF oscillations was high, and as the clinical course progressed, the HF oscillations were suppressed and the proportion of VLF oscillations and LF/HF_SKNA_ ratio were observed to increase.

In the SKNA analysis, compared with the control data, the acute phase showed significant enhancements of the non‐burst amplitude of aSKNA (2.4 [IQR: 1.3–4.1] vs. 0.7 [IQR: 0.5–1.7] μV, *p* < 0.001) (Figure 4a). The non‐burst and burst amplitudes of aSKNA reached a peak during the DCI phase and then gradually attenuated toward the post‐DCI phase. The acute phase showed a significantly decreased LF/HF_SKNA_ ratio compared to the control data (0.8 [IQR: 0.5–1.1] vs. 1.1 [IQR: 0.7–1.3], *p* = 0.028) (Figure 4f). The other frequency parameters in the three phases had no significant differences, compared with the control data. However, in the acute phase, the proportion of HF oscillations was high, and as the clinical course progressed, the HF oscillations were suppressed and the proportion of VLF oscillations and LF/HF_SKNA_ ratio increased.

**FIGURE 5:**
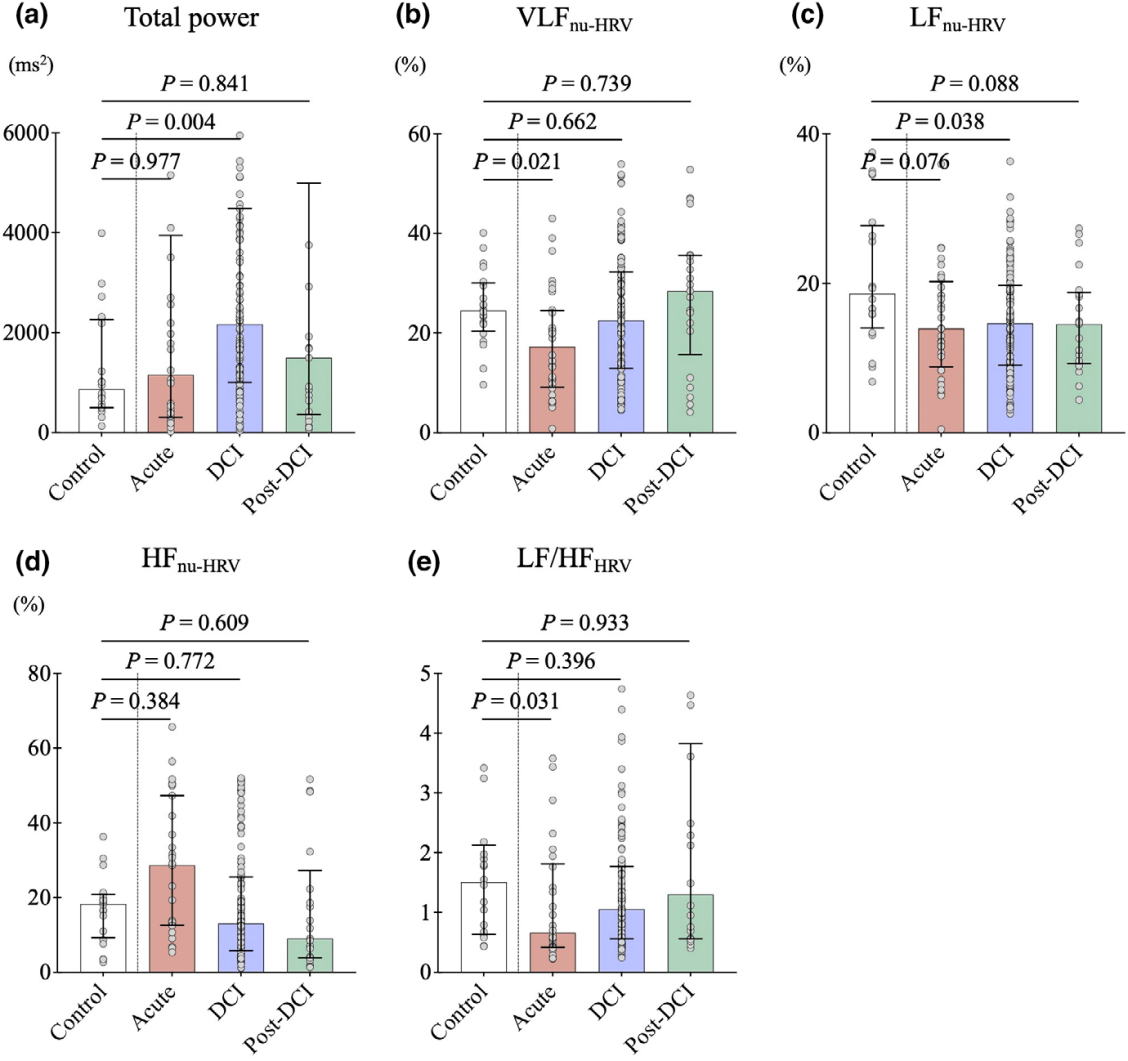
Effects of subarachnoid hemorrhage (SAH) on the heart rate variability (HRV) and its time course. The dots in the figure represent each HRV data, and the solid lines represent the median and quartiles. (a) HRV analysis revealed significant increase in total power during the delayed cerebral ischemia (DCI) phase (*n* = 127). (b) Very low frequency (VLF_nu‐HRV_) amplitude decreased during the acute phase and increased toward the post‐DCI phase (*n* = 21). (c) Low frequency (LF_nu‐HRV_) amplitude tended to decrease in entire phase. In the acute phase (*n* = 31), High frequency (HF_nu‐HRV_) amplitude was prominent (d) and the LF/HF_HRV_ ratio decreased markedly (e), however, toward the post‐DCI phase, HF_nu‐HRV_ decreased and conversely the LF/HF_HRV_ ratio increased.

HRV analysis revealed that compared with the control data (Figure 5), there was a significant decrease in the LF/HF_HRV_ ratio during the acute phase (0.6 [IQR: 0.4–1.4] vs. 1.5 [IQR: 0.6–2.1], *p* = 0.031) and a significant increase in total power during the DCI phase (2169 [IQR: 1001–4482] vs. 866 [IQR: 498–2260] ms^2^, *p* = 0.004). The transition of the frequency components during the clinical course was very similar to that of the SKNA, with the HF oscillations being noticeable during the acute phase and the VLF oscillations and LF/HF_HRV_ ratio increasing toward the post‐DCI phase.

### 3.4 Relationship between ECG alterations and the SKNA parameters

The relationship between the Tp‐e interval and SNA parameters was analyzed using a linear mixed model. Table 3 shows the degrees of freedom, standard error, *t* value, and *p* value of the relationship of SKNA and HRV parameters with the Tp‐e interval in the acute phase. No significant association was found for any parameter in the control data. In the SAH data, we observed a strong relationship between the non‐burst and burst amplitudes of aSKNA and the Tp‐e interval, especially in the acute phase. However, no association was observed in the DCI and post‐DCI phases. Although a similar analysis was performed for the QTc interval, there were no parameters with significant differences in any phase, and a relationship as strong with SNA as the Tp‐e interval was not shown.

**TABLE 3:** Estimates and *p* values for the fixed effects in linear mixed models.

	Value	SE	DF	*t* Value	*p* Value
Control					
(Intercept)	41.9	11.9	12.0	3.5	0.004
HR (bpm)	0.3	0.2	12.0	1.4	0.179
Non‐burst amplitude (μV)	−4.4	5.2	12.0	−0.9	0.414
Burst amplitude (μV)	2.7	3.7	12.0	0.7	0.483
LF/HF_SKNA_	−2.6	2.8	12.0	−0.9	0.360
LF/HF_HRV_	0.2	0.8	12.0	0.2	0.841
Acute phase					
(Intercept)	113.9	9.9	7.1	11.5	< 0.001
HR (bpm)	−0.6	0.1	6.2	−5.8	0.001
Non‐burst amplitude (μV)	−37.1	5.3	5.9	−7.0	<0.001
Burst amplitude (μV)	34.2	5.1	8.1	6.7	<0.001
LF/HF_SKNA_	−10.1	5.9	4.9	−1.7	0.148
LF/HF_HRV_	2.0	3.1	22.5	0.6	0.529
DCI phase					
(Intercept)	85.5	7.8	87.1	11.0	< 0.001
HR (bpm)	−0.1	0.1	103.4	−1.2	0.219
Non‐burst amplitude (μV)	0.1	1.4	110.7	0.1	0.949
Burst amplitude (μV)	−0.1	1.0	108.2	−0.1	0.926
LF/HF_SKNA_	−1.1	1.7	114.9	−0.7	0.506
LF/HF_HRV_	−0.8	0.7	114.7	−1.4	0.692
Post‐DCI phase					
(Intercept)	80.4	11.5	9.5	7.0	<0.001
HR (bpm)	−0.1	0.1	11.6	−0.9	0.411
Non‐burst amplitude (μV)	−0.5	4.5	11.9	−0.1	0.906
Burst amplitude (μV)	2.1	3.1	11.9	0.7	0.517
LF/HF_SKNA_	−9.5	4.7	8.3	−2.0	0.079
LF/HF_HRV_	−1.8	0.9	12.0	−1.9	0.085

Abbreviations: bpm, beats per minutes; DCI, delayed cerebral ischemia; DF, degree of freedom; HF, high frequency; HR, heart rate; HRV, heart rate variability; LF, low frequency; SE, standard error; SKNA, skin sympathetic nerve activity.

### 3.5 Comparison of the SKNA parameters between DCI and non‐DCI recordings

**FIGURE 6:**
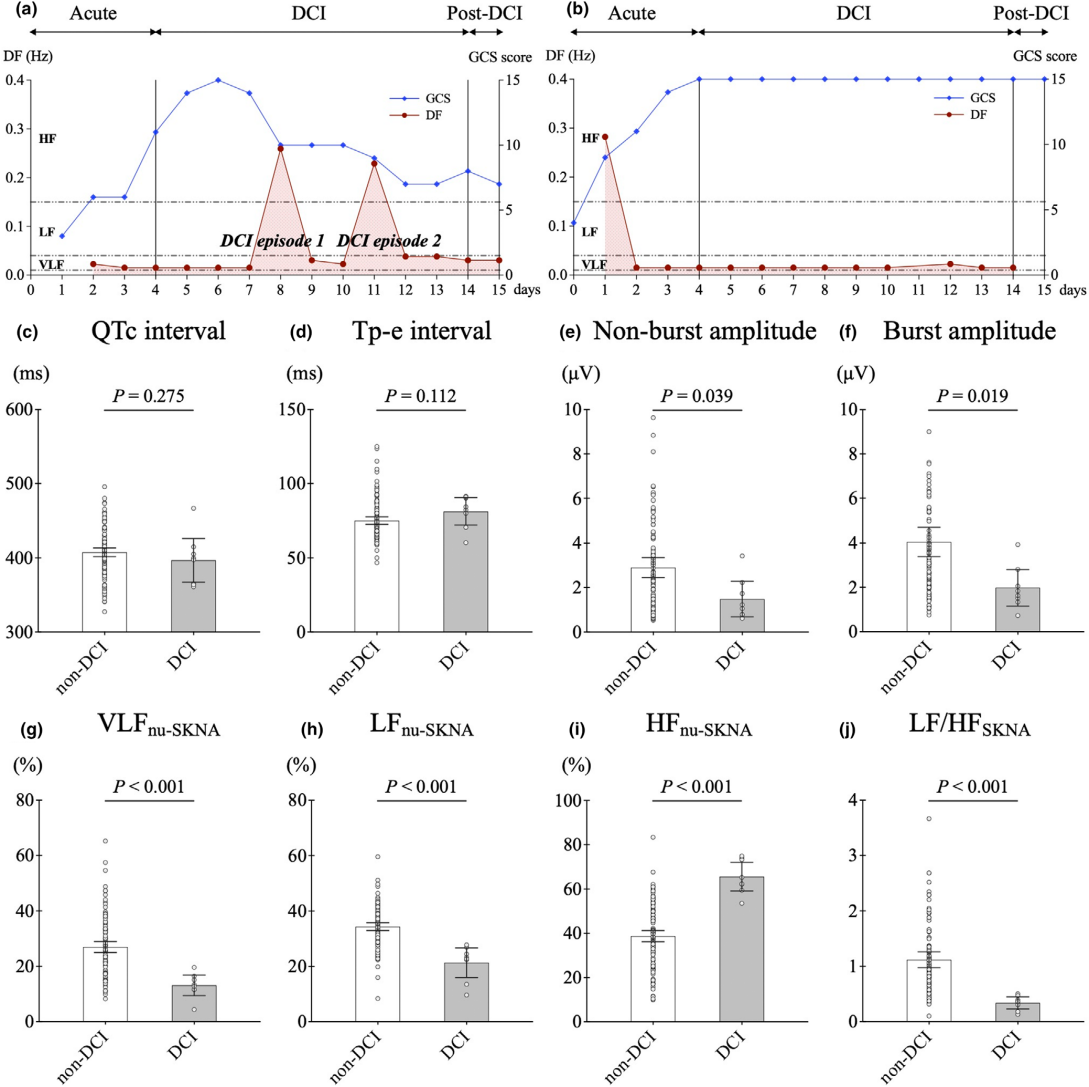
Comparison of skin sympathetic nerve activity (SKNA) parameters between delayed cerebral ischemia (DCI) and non‐DCI recordings. (a) In a representative clinical course of a case with DCI episodes, the Glasgow Coma Scale (GCS) is seen to initially improve but worsened with the occurrence of DCI episodes on days 8 and 11. At that time, the dominant frequency (DF) shifted from a very low frequency (VLF) to a high frequency (HF) oscillation. (b) In patients without DCI episodes, no shift in DF is observed. The dots in the figure represent each SKNA data in the DCI phase, and the solid lines represent the median and quartiles. Compared with the non‐DCI recordings (*n* = 104), the DCI recordings (*n* = 8) had no significant differences in (c) the corrected QT (QTc) and (d) the T peak‐to‐end (Tp‐e) intervals; significantly lower (e) non‐burst amplitude of average SKNA (aSKNA), (f) burst amplitude of aSKNA, (g) very low frequency (VLF_nu‐SKNA_), (h) low frequency (LF_nu‐SKNA_), and (i) ratio of low frequency to high frequency (LF/HF_SKNA_); and significantly higher predominance of (j) HF_nu‐SKNA_.

The DF shifts from VLF to HF oscillation were observed in 23 of 127 (18.1%) data within the DCI phase. Of the 23 data points that showed frequency shifts, eight were related to DCI episodes and were labeled as DCI recordings, two were related to a seizure episode, and two were related to hyperthermia. On the contrary, in patients without DCI episodes, there was no change in the DF during the DCI phase. The representative clinical course of cases with and without DCI episodes are shown in Figure 6. In a representative clinical course of a case with DCI episodes (Figure 6a), the GCS is seen to initially improve but worsened with the occurrence of DCI episodes on days 8 and 11. At that time, the DF shifted from a VLF to a HF oscillation. In patients without DCI episodes, no shift in DF is observed (Figure 6b). Figure 6c–j shows a comparison of ECG and SKNA parameters between non‐DCI (*n* = 104) and DCI recordings (*n* = 8). Compared with the non‐DCI recordings, the DCI recordings had no significant differences in the QTc and Tp‐e intervals; significantly lower non‐burst and burst amplitudes of aSKNA, VLF_nu‐SKNA_, LF_nu‐SKNA_, and LF/HF_SKNA_ ratio; and significantly higher predominance of HF_nu‐SKNA_.

### 3.6 Post hoc power of the study

Given the small sample size in this study, it is necessary to estimate if our study has adequate power to truly detect differences between SAH data and control data. When performing a Wilcoxon rank‐sum test for the Tp‐e interval between the acute phase of SAH and the control data, an effect size of 1.7 and a post hoc power of 0.9 were calculated using a significance level of 0.05. Similarly, for the burst amplitude of aSKNA comparison, an effect size of 0.8 and post hoc power of 0.7 were calculated. This shows that our sample size was adequate, and the study was sufficiently powered to truly detect differences.

## 4 DISCUSSION

### 4.1 Major findings

The primary findings of the present study were a significant aSKNA enhancement, a significant decrease in the LF/HF_SKNA_ and LF/HF_HRV_ ratio, and prolongation of the QTc and Tp‐e intervals during the acute phase of SAH. These findings support brain–heart interactions during the acute phase of SAH. Furthermore, in the human time course of SAH, we confirmed that enhancement of SNA and abnormalities in ECG repolarization simultaneously occurred from the acute phase, then gradually declined over the clinical course. The VLF_nu‐SKNA_ decreased and HF_nu‐SKNA_ became more pronounced when a DCI episode occurred.

### 4.2 ECG abnormalities and SAH

Previous studies reported that ECG alterations, particularly abnormalities in the repolarization process, were observed in more than 65% of SAH patients (Frontera et al., 2008). ECG alterations after SAH are important as prognostic factors for cardiomyopathy and arrhythmia events (Elsharkawy et al., 2016; Takenaka et al., 2006). However, the mechanism of ECG abnormalities is not well understood, and most reports focus on ECG abnormalities in the early stages after the onset of SAH, and there are no observational studies that have investigated ECG alterations over the clinical course. Similar to previous reports (Frontera et al., 2008), the present study found that in the acute phase of SAH, the QTc interval was significantly prolonged, and the PR interval remained unchanged. Although a previous report indicated a correlation between prolongation of the Tp‐e interval and neurological prognosis (Komatsuzaki et al., 2021), the present study did not observe reprolongation of the Tp‐e interval during a DCI event. Both QT and Tp‐e intervals have been recognized as indicators of repolarization process dispersion, with the Tp‐e interval as a measure of total myocardial repolarization dispersion (Opthof et al., 2007). The present study showed a relationship between the Tp‐e interval and SKNA, and the normalization process of the QTc interval was delayed. The Tp‐e interval may be more reflective of cardiac SNA.

### 4.3 Autonomic nervous system and SAH

Despite extensive research on the link between SAH and SNA, the underlying mechanisms remain unclear. Traditionally, intracranial hematoma compressing sympathetic centers like the hypothalamus was thought to trigger SNA overactivity (Hasegawa et al., 2022). Notably, past studies highlight the crucial role of post‐SAH catecholamine surges impacting organs like the heart, which can last for days (Dinh et al., 2020; Naredi et al., 2000). Logistical limitations and delayed results prevented continuous plasma catecholamine monitoring in our study.

SNA enhancement and catecholamine overproduction during the acute phase of SAH cause negative feedback to the cardiogenic centers of the brain (Borutta et al., 2022). This may lead to a temporary reduction in SNA tone and overall autonomic cardiovascular regulation, with a relative increase in parasympathetic activity. We observed an increase in aSKNA during the acute phase, peaking during the DCI phase, and gradually decreasing toward the post‐DCI phase. The frequency domain of SKNA and HRV showed similar trends, with the LF/HF ratio suppressed during the acute phase and normalized over time. Notably, DCI episodes disrupted this normalization process, causing a temporary dominance of HF oscillations and a decrease in the LF/HF ratio. These findings suggest that while SNA is enhanced during the acute phase of SAH, frequency analysis of SKNA and HRV hints at a potential predominance of parasympathetic activity.

Several studies have reported right‐sided stroke increased sympathetic cardiovascular regulation and arrhythmia (Colivicchi et al., 2004; Dütsch et al., 2007). In our study, the aneurysm rupture location and the affected hemisphere did not show a clear unilateral dominance. Due to the disease characteristics of SAH, the hemorrhage often extends throughout the entire cerebral cisterna, potentially compressing even the healthy side. This makes it difficult to determine the relationship between the affected hemisphere and SKNA changes in SAH. Further studies are needed to clarify this relationship.

### 4.4 Relationship between ECG alterations and SKNA after SAH

Under normal physiological conditions, SNA enhancement shortens the QT interval through increased outward potassium current. Contrary to this prediction, our study showed a relationship between SKNA enhancement and Tp‐e interval after SAH. Various factors have been proposed as causes of ECG abnormalities in SAH, including catecholamine‐induced cardiotoxicity (Samuels, 2007), electrolyte imbalance (Fukui et al., 2003), intracranial inflammation, and increased cerebral pressure due to hematoma (Lorenzo et al., 1994). Additionally, brain–heart interactions via SNA dysregulation are believed to play a role (Samuels, 2007). Sympathetic stimulation can prolong the QT interval due to dysfunction of the slowly activating delayed rectifier potassium current (IKs), such as drug‐induced or congenital long QT syndrome type 1 (Shimizu & Antzelevitch, 1998). However, none of our patients were known to have congenital long QT syndrome or were receiving IKs blockers. It is unlikely that dysfunction of IKs explained our unexpected observations. We hypothesize that a temporary breakdown of the sympathetic nerve regulatory function occurs during the acute phase of SAH. This transient dysregulation of SNA is the most likely mechanism underlying the observed ECG alterations. Experimental models using canine HRV parameters have shown that the QT interval prolongs when the LF/HF ratio decreases (Harada et al., 2005). In our study, ECG alterations also improved with normalization of SKNA parameters, suggesting a relationship with sympathetic tone and autonomic regulation.

### 4.5 Potential for the clinical application of neuECG

neuECG is a unique tool that is capable of simultaneous, noninvasive, and continuous measurements of ECG and SKNA. The ability to measure without special skills and independent of patient condition overcomes the problems of traditional SNA assessment tools and expands opportunities for clinical application. As an example, we observed a frequency shift from VLF to HF oscillations in all cases during DCI recordings. Although various tests, such as CT angiography, MRI, transcranial doppler ultrasonography, and electroencephalography, are effective in evaluating DCI, early detection has been difficult (Balança et al., 2018; Kumar et al., 2016; Mir et al., 2014; Sastry et al., 2022). We propose the effectiveness of neuECG as a monitoring tool in SAH and plan further studies to evaluate its effectiveness and accuracy.

### 4.6 Study limitations

The present study is limited by its single‐center design and predominantly Asian participants. External factors, such as body position, temperature, mechanical ventilation, and medication use may have influenced SNA (Pöyhönen et al., 2004). Additionally, the study did not comprehensively evaluate autonomic nervous system, including sympathetic afferent pathway, parasympathetic activity, and serum biomarkers. The 30 min daily recording period limited our ability to assess circadian rhythm effects on SNA. Since this observational study mainly aimed to confirm the possibility of clinical application of neuECG, relationship between SKNA and other modalities for SNA related to skin response (e.g., skin blood flow and sweat rate) have not been sufficiently investigated. Future studies should employ approaches to comprehensively assess autonomic nervous system, including larger and more diverse populations and long‐term monitoring to capture circadian variation.

## 5 CONCLUSIONS

Transient sympathetic dysregulation contributes to repolarization impairment that in turn could cause arrhythmias. neuECG recordings may have the potential of a noninvasive method to monitor adverse outcomes in patients with SAH.

## AUTHOR CONTRIBUTIONS

TK and ST contributed to the concept and design of the study. ST and YN contributed to data collection and analysis. YN contributed to the first draft of the article. All authors contributed to the critical revision and approval of the manuscript.

## FUNDING INFORMATION

The authors received no financial support for the research, authorship, and for the publication of this article.

## CONFLICT OF INTEREST STATEMENT

The authors report no conflicts of interest and that the funding sources did not play a role in the design, collection, analysis and interpretation of the data, in the writing of the manuscript and the decision to submit the manuscript for publication.

## Data Availability

The data sets used and/or analyzed during the present study are available from the corresponding author on reasonable request.

## References

[phy270202-bib-0001] Balança, B. , Dailler, F. , Boulogne, S. , Ritzenthaler, T. , Gobert, F. , Rheims, S. , & Andre‐Obadia, N. (2018). Diagnostic accuracy of quantitative EEG to detect delayed cerebral ischemia after subarachnoid hemorrhage: A preliminary study. Clinical Neurophysiology, 129(9), 1926–1936. 10.1016/j.clinph.2018.06.013 30007892

[phy270202-bib-0002] Battaglini, D. , Robba, C. , Lopes da Silva, A. , Dos Santos Samary, C. , Leme Silva, P. , Dal Pizzol, F. , Pelosi, P. , & Rocco, P. R. M. (2020). Brain‐heart interaction after acute ischemic stroke. Critical Care, 24(1), 163. 10.1186/s13054-020-02885-8 32317013 PMC7175494

[phy270202-bib-0003] Borutta, M. C. , Gerner, S. T. , Moeser, P. , Hoelter, P. , Engelhorn, T. , Doerfler, A. , Huttner, H. B. , Schwab, S. , Kuramatsu, J. B. , & Koehn, J. (2022). Correlation between clinical severity and extent of autonomic cardiovascular impairment in the acute phase of subarachnoid hemorrhage. Journal of Neurology, 269(10), 5541–5552. 10.1007/s00415-022-11220-w 35723723 PMC9467940

[phy270202-bib-0004] Chen, M. , Wang, Z. , Lai, X. , Wang, S. , Wu, Z. , Liu, Q. , & Zhou, S. (2023). Transient cardiac electrophysiological changes in a rat model of subarachnoid haemorrhage: A brain‐heart interaction. Europace, 25(6), euad171. 10.1093/europace/euad171 37337928 PMC10306271

[phy270202-bib-0005] Colivicchi, F. , Bassi, A. , Santini, M. , & Caltagirone, C. (2004). Cardiac autonomic derangement and arrhythmias in right‐sided stroke with insular involvement. Stroke, 35(9), 2094–2098. 10.1161/01.str.0000138452.81003.4c 15272134

[phy270202-bib-0006] Demura, M. , Ishii, H. , Takarada‐Iemata, M. , Kamide, T. , Yoshikawa, A. , Nakada, M. , & Hori, O. (2023). Sympathetic nervous hyperactivity impairs microcirculation, leading to early brain injury after subarachnoid hemorrhage. Stroke, 54(6), 1645–1655. 10.1161/STROKEAHA.123.042799 37154061

[phy270202-bib-0007] Dinh, D. D. , Lidington, D. , Kroetsch, J. T. , Ng, C. , Zhang, H. , Nedospasov, S. A. , Heximer, S. P. , & Bolz, S. S. (2020). Experimental subarachnoid hemorrhage drives catecholamine‐dependent cardiac and peripheral microvascular dysfunction. Frontiers in Physiology, 11, 402. 10.3389/fphys.2020.00402 32477159 PMC7237757

[phy270202-bib-0008] Doytchinova, A. , Hassel, J. L. , Yuan, Y. , Lin, H. , Yin, D. , Adams, D. , Straka, S. , Wright, K. , Smith, K. , Wagner, D. , Shen, C. , Salanova, V. , Meshberger, C. , Chen, L. S. , Kincaid, J. C. , Coffey, A. C. , Wu, G. , Li, Y. , Kovacs, R. J. , … Chen, P. S. (2017). Simultaneous noninvasive recording of skin sympathetic nerve activity and electrocardiogram. Heart Rhythm, 14(1), 25–33. 10.1016/j.hrthm.2016.09.019 27670627 PMC5182108

[phy270202-bib-0009] Dütsch, M. , Burger, M. , Dörfler, C. , Schwab, S. , & Hilz, M. J. (2007). Cardiovascular autonomic function in poststroke patients. Neurology, 69(24), 2249–2255. 10.1212/01.wnl.0000286946.06639.a7 18071145

[phy270202-bib-0010] Elsharkawy, H. , Abd‐Elsayed, A. , El‐Hadi, S. , Provencio, J. , & Tetzlaff, J. (2016). Fluctuating electrocardiographic changes predict poor outcomes after acute subarachnoid hemorrhage. The Ochsner Journal, 16(3), 225–229. 27660569 PMC5024802

[phy270202-bib-0011] Frontera, J. A. , Parra, A. , Shimbo, D. , Fernandez, A. , Schmidt, J. M. , Peter, P. , Claassen, J. , Wartenberg, K. E. , Rincon, F. , Badjatia, N. , Naidech, A. , Connolly, E. S. , & Mayer, S. A. (2008). Cardiac arrhythmias after subarachnoid hemorrhage: Risk factors and impact on outcome. Cerebrovascular Diseases, 26(1), 71–78. 10.1159/000135711 18525201 PMC2909703

[phy270202-bib-0012] Fukui, S. , Katoh, H. , Tsuzuki, N. , Ishihara, S. , Otani, N. , Ooigawa, H. , Toyooka, T. , Ohnuki, A. , Miyazawa, T. , Nawashiro, H. , & Shima, K. (2003). Multivariate analysis of risk factors for QT prolongation following subarachnoid hemorrhage. Critical Care, 7(3), R7–R12. 10.1186/cc2160 12793884 PMC270671

[phy270202-bib-0013] Harada, T. , Abe, J. , Shiotani, M. , Hamada, Y. , & Horii, I. (2005). Effect of autonomic nervous function on QT interval in dogs. The Journal of Toxicological Sciences, 30(3), 229–237. 10.2131/jts.30.229 16141656

[phy270202-bib-0014] Hart, E. C. , Head, G. A. , Carter, J. R. , Wallin, B. G. , May, C. N. , Hamza, S. M. , Hall, J. E. , Charkoudian, N. , & Osborn, J. W. (2017). Recording sympathetic nerve activity in conscious humans and other mammals: Guidelines and the road to standardization. American Journal of Physiology. Heart and Circulatory Physiology, 312(5), H1031–H1051. 10.1152/ajpheart.00703.2016 28364017 PMC6146303

[phy270202-bib-0015] Hasegawa, Y. , Uchikawa, H. , Kajiwara, S. , & Morioka, M. (2022). Central sympathetic nerve activation in subarachnoid hemorrhage. Journal of Neurochemistry, 160(1), 34–50. 10.1111/jnc.15511 34525222

[phy270202-bib-0016] Jain, V. , Rath, G. P. , Dash, H. H. , Bithal, P. K. , Chouhan, R. S. , & Suri, A. (2011). Stellate ganglion block for treatment of cerebral vasospasm in patients with aneurysmal subarachnoid hemorrhage—A preliminary study. Journal of Anaesthesiology Clinical Pharmacology, 27(4), 516–521. 10.4103/0970-9185.86598 22096287 PMC3214559

[phy270202-bib-0017] Jiang, Z. , Zhao, Y. , Doytchinova, A. , Kamp, N. J. , Tsai, W. C. , Yuan, Y. , Adams, D. , Wagner, D. , Shen, C. , Chen, L. S. , Everett, T. H., 4th , Lin, S. F. , & Chen, P. S. (2015). Using skin sympathetic nerve activity to estimate stellate ganglion nerve activity in dogs. Heart Rhythm, 12(6), 1324–1332. 10.1016/j.hrthm.2015.02.012 25681792 PMC4442039

[phy270202-bib-0018] Kawasaki, T. , Azuma, A. , Sawada, T. , Sugihara, H. , Kuribayashi, T. , Satoh, M. , Shimizu, Y. , & Nakagawa, M. (2002). Electrocardiographic score as a predictor of mortality after subarachnoid hemorrhage. Circulation Journal, 66(6), 567–570. 10.1253/circj.66.567 12074275

[phy270202-bib-0019] Kligfield, P. , & Okin, P. M. (2007). Prevalence and clinical implications of improper filter settings in routine electrocardiography. The American Journal of Cardiology, 99(5), 711–713. 10.1016/j.amjcard.2006.09.123 17317378

[phy270202-bib-0020] Komatsuzaki, M. , Takasusuki, T. , Kimura, Y. , & Yamaguchi, S. (2021). Assessment of the ECG T‐wave in patients with subarachnoid hemorrhage. Journal of Neurosurgical Anesthesiology, 33(1), 58–64. 10.1097/ANA.0000000000000624 31290770

[phy270202-bib-0021] Komi, P. V. , & Tesch, P. (1979). EMG frequency spectrum, muscle structure, and fatigue during dynamic contractions in man. European Journal of Applied Physiology and Occupational Physiology, 42(1), 41–50. 10.1007/BF00421103 499196

[phy270202-bib-0022] Kumar, G. , Shahripour, R. B. , & Harrigan, M. R. (2016). Vasospasm on transcranial doppler is predictive of delayed cerebral ischemia in aneurysmal subarachnoid hemorrhage: A systematic review and meta‐analysis. Journal of Neurosurgery, 124(5), 1257–1264. 10.3171/2015.4.JNS15428 26495942

[phy270202-bib-0023] Kusayama, T. , Wong, J. , Liu, X. , He, W. , Doytchinova, A. , Robinson, E. A. , Adams, D. E. , Chen, L. S. , Lin, S. F. , Davoren, K. , Victor, R. G. , Cai, C. , Dai, M. Y. , Tian, Y. , Zhang, P. , Ernst, D. , Rho, R. H. , Chen, M. , Cha, Y. M. , … Chen, P. S. (2020). Simultaneous noninvasive recording of electrocardiogram and skin sympathetic nerve activity (neuECG). Nature Protocols, 15(5), 1853–1877. 10.1038/s41596-020-0316-6 32313253

[phy270202-bib-0024] Liu, X. , Yuan, Y. , Wong, J. , Meng, G. , Ueoka, A. , Woiewodski, L. M. , Chen, L. S. , Shen, C. , Li, X. , Lin, S. F. , Everett, T. H., 4th , & Chen, P. S. (2021). The frequency spectrum of sympathetic nerve activity and arrhythmogenicity in ambulatory dogs. Heart Rhythm, 18(3), 465–472. 10.1016/j.hrthm.2020.11.023 33246037

[phy270202-bib-0025] Lorenzo, N. Y. , Earle, A. M. , Peterson, L. L. , Todd, G. L. , & Leilbrock, L. G. (1994). The relationship of the subarachnoid injection of blood and blood fractions with cardiac rate change and arrhythmias. Journal of the Neurological Sciences, 127(2), 134–142. 10.1016/0022-510x(94)90065-5 7707072

[phy270202-bib-0026] Marion, D. W. , Segal, R. , & Thompson, M. E. (1986). Subarachnoid hemorrhage and the heart. Neurosurgery, 18(1), 101–106. 10.1227/00006123-198601000-00019 3511399

[phy270202-bib-0027] Meng, G. , He, W. , Wong, J. , Li, X. , Mitscher, G. A. , Straka, S. , Adams, D. , Everett, T. H., 4th , Manchanda, S. , Liu, X. , Chen, P. S. , & Tang, Y. (2022). Successful continuous positive airway pressure treatment reduces skin sympathetic nerve activity in patients with obstructive sleep apnea. Heart Rhythm, 19(1), 127–136. 10.1016/j.hrthm.2021.09.018 34562644 PMC8742760

[phy270202-bib-0028] Mir, D. I. , Gupta, A. , Dunning, A. , Puchi, L. , Robinson, C. L. , Epstein, H. A. , & Sanelli, P. C. (2014). CT perfusion for detection of delayed cerebral ischemia in aneurysmal subarachnoid hemorrhage: A systematic review and meta‐analysis. AJNR. American Journal of Neuroradiology, 35(5), 866–871. 10.3174/ajnr.A3787 24309123 PMC4159608

[phy270202-bib-0029] Naredi, S. , Lambert, G. , Edén, E. , Zäll, S. , Runnerstam, M. , Rydenhag, B. , & Friberg, P. (2000). Increased sympathetic nervous activity in patients with nontraumatic subarachnoid hemorrhage. Stroke, 31(4), 901–906. 10.1161/01.str.31.4.901 10753996

[phy270202-bib-0030] Opthof, T. , Coronel, R. , Wilms‐Schopman, F. J. , Plotnikov, A. N. , Shlapakova, I. N. , Danilo, P., Jr. , Rosen, M. R. , & Janse, M. J. (2007). Dispersion of repolarization in canine ventricle and the electrocardiographic T wave: Tp‐e interval does not reflect transmural dispersion. Heart Rhythm, 4(3), 341–348. 10.1016/j.hrthm.2006.11.022 17341400

[phy270202-bib-0031] Pöyhönen, M. , Syväoja, S. , Hartikainen, J. , Ruokonen, E. , & Takala, J. (2004). The effect of carbon dioxide, respiratory rate and tidal volume on human heart rate variability. Acta Anaesthesiologica Scandinavica, 48(1), 93–101. 10.1111/j.1399-6576.2004.00272.x 14674979

[phy270202-bib-0032] Roos, Y. B. , de Haan, R. J. , Beenen, L. F. , Groen, R. J. , Albrecht, K. W. , & Vermeulen, M. (2000). Complications and outcome in patients with aneurysmal subarachnoid haemorrhage: A prospective hospital based cohort study in The Netherlands. Journal of Neurology, Neurosurgery, and Psychiatry, 68(3), 337–341. 10.1136/jnnp.68.3.337 10675216 PMC1736841

[phy270202-bib-0033] Samuels, M. A. (2007). The brain‐heart connection. Circulation, 116(1), 77–84. 10.1161/circulationaha.106.678995 17606855

[phy270202-bib-0034] Sastry, R. A. , Bajaj, A. , Shaaya, E. A. , Anderson, M. N. , & Doberstein, C. (2022). Utility of automated MRI perfusion (RAPID) with or without MR angiography for detection of angiographic vasospasm after aneurysmal subarachnoid hemorrhage. Journal of Clinical Neuroscience, 100, 143–147. 10.1016/j.jocn.2022.04.021 35468351

[phy270202-bib-0035] Shimizu, W. , & Antzelevitch, C. (1998). Cellular basis for the ECG features of the LQT1 form of the long‐QT syndrome: Effects of beta‐adrenergic agonists and antagonists and sodium channel blockers on transmural dispersion of repolarization and torsade de pointes. Circulation, 98(21), 2314–2322. 10.1161/01.cir.98.21.2314 9826320

[phy270202-bib-0036] Takenaka, I. , Aoyama, K. , Iwagaki, T. , Ishimura, H. , & Kadoya, T. (2006). Development of torsade de pointes caused by exacerbation of QT prolongation during clipping of cerebral artery aneurysm in a patient with subarachnoid haemorrhage. British Journal of Anaesthesia, 97(4), 533–535. 10.1093/bja/ael183 16849385

[phy270202-bib-0037] Taniguchi, T. , Morimoto, M. , Taniguchi, Y. , Takasaka, M. , & Totoki, T. (1994). Cutaneous distribution of sympathetic postganglionic fibers from stellate ganglion: A retrograde axonal tracing study using wheat germ agglutinin conjugated with horseradish peroxidase. Journal of Anesthesia, 8(4), 441–449. 10.1007/BF02514624 28921353

[phy270202-bib-0038] Tiwari, R. , Kumar, R. , Malik, S. , Raj, T. , & Kumar, P. (2021). Analysis of heart rate variability and implication of different factors on heart rate variability. Current Cardiology Reviews, 17(5), e160721189770. 10.2174/1573403x16999201231203854 33390146 PMC8950456

[phy270202-bib-0039] Vergouwen, M. D. , Vermeulen, M. , van Gijn, J. , Rinkel, G. J. , Wijdicks, E. F. , Muizelaar, J. P. , Mendelow, A. D. , Juvela, S. , Yonas, H. , Terbrugge, K. G. , Macdonald, R. L. , Diringer, M. N. , Broderick, J. P. , Dreier, J. P. , & Roos, Y. B. (2010). Definition of delayed cerebral ischemia after aneurysmal subarachnoid hemorrhage as an outcome event in clinical trials and observational studies: Proposal of a multidisciplinary research group. Stroke, 41(10), 2391–2395. 10.1161/STROKEAHA.110.589275 20798370

